# Supplementary figures and images for: A comparative study of dengue virus vectors in major parks and adjacent residential areas in Ho Chi Minh City, Vietnam
Source: PLoS Negl Trop Dis. 2022 Jan 12;16(1):e0010119. doi: 10.1371/journal.pntd.0010119 (PMC8789112; doi:10.1371/journal.pntd.0010119)

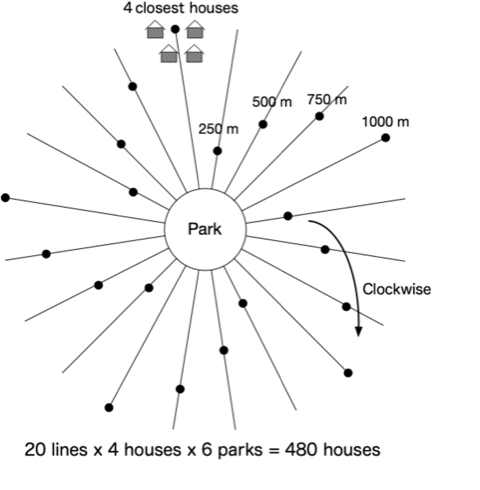

Supplement: S1 Fig — (TIFF) [file pntd.0010119.s001.tiff]

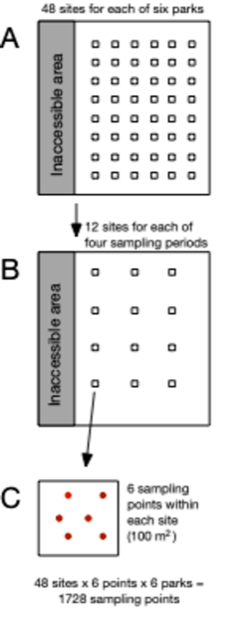

Supplement: S2 Fig — (A) 48 sampling sites in each park, (B) 12 sites for each of four sampling periods in two days, and (C) six sampling points at each sapling site. (TIFF) [file pntd.0010119.s002.tiff]

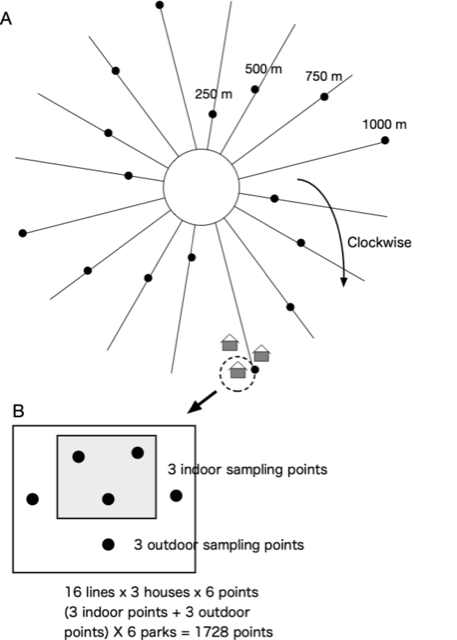

Supplement: S3 Fig — (A) three closest houses at each of 16 sampling sites at four different distances from the parks, and (B) six sampling points within each house. (TIFF) [file pntd.0010119.s003.tiff]
