# Supplementary material for: A comparative study of dengue virus vectors in major parks and adjacent residential areas in Ho Chi Minh City, Vietnam
Source: PLoS Negl Trop Dis. 2022 Jan 12;16(1):e0010119. doi: 10.1371/journal.pntd.0010119 (PMC8789112; doi:10.1371/journal.pntd.0010119)
Supplement: Attachment — Submitted filename: PNTD-D-21-01057 (1).pdf [file pntd.0010119.s007.pdf]

PLOS Neglected Tropical Diseases

A comparative study of dengue virus vectors in major parks and adjacent residential areas in Ho Chi Minh City, Vietnam

--Manuscript Draft--

**Manuscript Number:**	PNTD-D-21-01057
**Full Title:**	A comparative study of dengue virus vectors in major parks and adjacent residential areas in Ho Chi Minh City, Vietnam
**Short Title:**	Dengue virus vectors in major parks in Ho Chi Minh City
**Article Type:**	Research Article
**Keywords:**	Aedes aegypti, Aedes albopictus, park, habitat, density, dengue, vector
**Abstract:**	The primary dengue virus vectors, Aedes aegypti and Aedes albopictus, are daytime biting mosquitoes. The risk of infection is suspected to be considerable in urban parks due to visitor traffic. Despite the importance of vector control for reducing dengue transmission, little information is available on vector populations in urban parks. The present study characterized mosquito habitats and estimated vector densities in the major urban parks in Ho Chi Minh City, Vietnam and compared them with those in adjacent residential areas. The proportions of habitats where Aedes larvae were found were 43% and 9% for the parks and residential areas, respectively. The difference was statistically significant (PR: 5.00, 95% CI: 3.85-6.49). The proportions of positive larval habitats were significantly greater in the parks for both species (PR: 1.52, 95% CI: 1.04-2.22 for A. aegypti, PR: 10.10, 95% CI: 7.23-14.12 for A. albopictus). Larvae of both species were positively associated with discarded containers and planters. Aedes albopictus larvae were negatively associated with indoor habitats, but positively associated with vegetation shade. The adult density of A. aegypti was significantly less in the parks compared with the residential areas (PR; 0.09, 95% CI: 0.05-0.16), while the density of A. albopictus was significantly higher in the parks (PR: 9.99, 95% CI: 6.85-14.59). When the species were combined, the density was significantly higher in the parks (PR: 2.50, 95% CI: 1.92-3.25). The urban parks provide suitable environment for Aedes mosquitoes, and A. albopictus in particular. Virus vectors are abundant in the urban parks, and the current vector control programs need to have greater consideration of urban parks.
**Additional Information:**	
**Question**	**Response**
**Financial Disclosure**<br><br>Enter a financial disclosure statement that describes the sources of funding for the work included in this submission. Review the submission guidelines for detailed requirements. View published research articles from *PLOS NTDs* for specific examples.<br><br>This statement is required for submission and **will appear in the published article** if the submission is accepted. Please make sure it is accurate.	The first author (TH) is grateful for the academic support from the Program for Nurturing Global Leaders in Tropical and Emerging Communicable Diseases, Graduate School of Biomedical Sciences, Nagasaki University. This research was supported by the Japan Agency for Medical Research and Development (AMED) under Grant Number JP21wm0125006. The funders had no role in study design, data collection and analysis, decision to publish, or preparation of the manuscript. The funders had no role in study design, data collection and analysis, decision to publish, or preparation of the manuscript.

**Unfunded studies**<br><br>Enter: *The author(s) received no specific funding for this work.*<br><br>**Funded studies**<br><br>Enter a statement with the following details:<br>• Initials of the authors who received each award<br>• Grant numbers awarded to each author<br>• The full name of each funder<br>• URL of each funder website<br>• Did the sponsors or funders play any role in the study design, data collection and analysis, decision to publish, or preparation of the manuscript?<br>• **NO** - Include this sentence at the end of your statement: *The funders had no role in study design, data collection and analysis, decision to publish, or preparation of the manuscript.*<br>• **YES** - Specify the role(s) played.<br><br>* typeset	
**Competing Interests**<br><br>Use the instructions below to enter a competing interest statement for this submission. On behalf of all authors, disclose any competing interests that could be perceived to bias this work—acknowledging all financial support and any other relevant financial or non-financial competing interests.<br><br>This statement **will appear in the published article** if the submission is accepted. Please make sure it is accurate. View published research articles from *PLOS NTDs* for specific examples.	The authors have declared that no competing interests exist.

Powered by Editorial Manager® and ProduXion Manager® from Aries Systems Corporation

**NO authors have competing interests**<br><br>Enter: *The authors have declared that no competing interests exist.*<br><br>**Authors with competing interests**<br><br>Enter competing interest details beginning with this statement:<br><br>*I have read the journal's policy and the authors of this manuscript have the following competing interests: [insert competing interests here]*<br><br>* typeset<br><br>This statement is required for submission and **will appear in the published article** if the submission is accepted. Please make sure it is accurate and that any funding sources listed in your Funding Information later in the submission form are also declared in your Financial Disclosure statement.	
**Data Availability**<br><br>Authors are required to make all data underlying the findings described fully available, without restriction, and from the time of publication. PLOS allows rare exceptions to address legal and ethical concerns. See the PLOS Data Policy and FAQ for detailed information.	No - some restrictions will apply

A Data Availability Statement describing where the data can be found is required at submission. Your answers to this question constitute the Data Availability Statement and **will be published in the article**, if accepted.<br><br>**Important:** Stating 'data available on request from the author' is not sufficient. If your data are only available upon request, select 'No' for the first question and explain your exceptional situation in the text box.<br><br>Do the authors confirm that all data underlying the findings described in their manuscript are fully available without restriction?	
**Describe where the data may be found in full sentences. If you are copying our sample text, replace any instances of XXX with the appropriate details.**<br><br>• If the data are **held or will be held in a public repository**, include URLs, accession numbers or DOIs. If this information will only be available after acceptance, indicate this by ticking the box below. For example: *All XXX files are available from the XXX database (accession number(s) XXX, XXX.).*<br>• If the data are all contained **within the manuscript and/or Supporting Information files**, enter the following: *All relevant data are within the manuscript and its Supporting Information files.*<br>• If neither of these applies but you are able to provide **details of access elsewhere**, with or without limitations, please do so. For example:<br><br>*Data cannot be shared publicly because*	The data that support the findings of this study are available from the corresponding author upon reasonable request.

Powered by Editorial Manager® and ProduXion Manager® from Aries Systems Corporation

of [XXX]. Data are available from the XXX Institutional Data Access / Ethics Committee (contact via XXX) for researchers who meet the criteria for access to confidential data.<br><br>*The data underlying the results presented in the study are available from (include the name of the third party and contact information or URL).*<br>• This text is appropriate if the data are owned by a third party and authors do not have permission to share the data.<br><br>* typeset	
Additional data availability information:	Tick here if the URLs/accession numbers/DOIs will be available only after acceptance of the manuscript for publication so that we can ensure their inclusion before publication.

Manuscript Click here to access/download;Manuscript;MS.docx

Research article for PLoS Neglected Tropical Diseases

Short title: Dengue virus vectors in major parks in Ho Chi Minh City

A comparative study of dengue virus vectors in major parks and adjacent residential areas in Ho Chi Minh City, Vietnam

Trang Huynh Thi Thuy[1,2,3], Noboru Minakawa[2*]

[1] Department of Medical Entomology and Zoonotics, Pasteur Institute in Ho Chi Minh City, Ho Chi Minh City, Vietnam

[2] Institute of Tropical Medicine (NEKKEN), Nagasaki University, Nagasaki, Japan

[3] Graduate School of Biomedical Sciences, Nagasaki University, Nagasaki, Japan

[*]Corresponding author: Noboru Minakawa

E-mail: minakawa@nagasaki-u.ac.jp

Abstract

The primary dengue virus vectors, *Aedes aegypti* and *Aedes albopictus,* are daytime biting mosquitoes. The risk of infection is suspected to be considerable in urban parks due to visitor traffic. Despite the importance of vector control for reducing dengue transmission, little information is available on vector populations in urban parks. The present study characterized mosquito habitats and estimated vector densities in the major urban parks in Ho Chi Minh City, Vietnam and compared them with those in adjacent residential areas. The proportions of habitats where *Aedes* larvae were found were 43% and 9% for the parks and residential areas, respectively. The difference was statistically significant (PR: 5.00, 95% CI: 3.85-6.49). The proportions of positive larval habitats were significantly greater in the parks for both species (PR: 1.52, 95% CI: 1.04-2.22 for *A. aegypti*, PR: 10.10, 95% CI: 7.23-14.12 for *A. albopictus*). Larvae of both species were positively associated with discarded containers and planters. *Aedes albopictus* larvae were negatively associated with indoor habitats, but positively associated with vegetation shade. The adult density of *A. aegypti* was significantly less in the parks compared with the residential areas (PR; 0.09, 95% CI: 0.05-0.16), while the density of *A. albopictus* was significantly higher in the parks (PR: 9.99, 95% CI: 6.85-14.59). When the species were combined, the density was significantly higher in the parks (PR: 2.50, 95% CI: 1.92-3.25). The urban parks provide suitable environment for *Aedes* mosquitoes, and *A. albopictus* in particular. Virus vectors are abundant in the urban parks, and the current vector control programs need to have greater consideration of urban parks.

Author summary

The primary dengue virus vectors, *Aedes aegypti* and *Aedes albopictus,* are daytime biting mosquitoes and therefore the risk of infection may be considerable in urban parks due to human foot traffic. Prior to the present study little information was available on vector populations in urban parks. Here we describe that larvae of both species were positively

associated with discarded containers and planters. *Aedes albopictus* larvae were negatively associated with indoor habitats, but positively associated with vegetation shade. *Aedes albopictus* was predominant in the urban parks while *A. aegypti* was predominant in adjacent residential areas. When the species were combined the density of vectors was greater in the urban parks. The current vector control programs need to take into consideration vector intensity within urban parks.

Introduction

According to the WHO, over the last two decades 100 to 400 million dengue virus infections occurred annually, and the number of cases increased eight-fold [1]. Dengue is primarily an urban disease, and the recent increase is likely associated with the rapid expansion of urban areas [2]. Between the years 2000 and 2015 over 70% of outbreaks occurred in urban areas or across a combination of urban and rural areas [3]. The dense human populations of urban areas increase vector-human contact, and dengue virus (DENV) circulates more efficiently [2]. However, recent seroprevalence studies showed that rural populations have been exposed to DENV as much as urban populations [4,5]. The high seropositive rate in rural areas is likely due to an increase of mobile populations and transitions toward urban areas.

The principal DENV vector species, *Aedes aegypti* and *Aedes albopictus*, are well adapted to urban environments. Larvae of both species are often found in discarded containers in residential areas with poor sanitation [6–9]. This is one of the reasons why high infection risk is often associated with socio-economic factors and rapid urbanization without proper infrastructure [2]. As a result, the abundance of vectors increases with urban sprawl in tropical regions. While the former species breeds almost exclusively in small anthropogenic habitats in urban areas [10], the latter species may also breed in tree holes and cut or broken bamboos [11].

Both vector species actively bite in the daytime. Because people may spend much of the day away from home, the risk of infection is not limited to residential areas. In the case of a dengue outbreak in Tokyo in 2014, nearly all infected persons had visited the same parks during the epidemic [12], and the populations of *A. albopictus* in the parks had extremely high biting densities and infection rates [13,14]. Numerous discarded containers are usually present in urban parks, and vegetation provides a suitable environment for *A. albopictus*

[15,16]. Although the scale of the outbreak in Tokyo was much smaller compared with epidemics in tropical areas, the incident implies that urban parks may also facilitate intense transmission in endemic regions, because many people of all age classes visit parks during the daytime when the vectors are active.

Although *A. albopictus* is the only dengue virus vector in Japan, both *A. aegypti* and *A. albopictus* are present in tropical and sub-tropical Asia. While *Ae. albopictus* is associated more with vegetation or rural areas [15–18], *A. aegypti* is predominant in large cities such as Ho Chi Minh City, Singapore, and Yangon [19–22]. Despite the potential risk of infection in urban parks, little information is available for the vector populations in urban parks except for a study in São Paulo, Brazil [7].

The present study addressed four hypotheses comparing the populations of *A. albopictus* and *A. aegypti* in the major parks and the adjacent residential areas in Ho Chi Minh City, Vietnam. Specifically: 1) the relative abundance of *A. albopictus* adults is greater in the parks compared with *Ae. aegypti* because of the greater vegetation cover, 2) more natural breeding habitats are available in the parks, 3) the preference of breeding habitat types is different between the two mosquito species, and 4) the abundance of both vector species in the parks is comparable to that in the residential areas. Evaluating hypotheses 1 to 3 provide important information for future vector control strategies, and hypothesis 4 assesses the infection risk in urban parks.

Methods

Study sites

Ho Chi Minh City is the most populous city in Vietnam with 8.9 million inhabitants within 2,061 km^2 in 2019. High dengue virus transmission mainly occurs during the rainy season in

the second half of the year [22]. Dengue morbidity and mortality increased in recent years, and the outbreak in 2017 numbered 33,729 cases in the city [23]. The city has approximately 210 parks, and the present study focused on the 6 largest and most popular parks (Gia Dinh, Le Thi Rieng, Phu Lam, Tao Dan, Thao Cam Vien, and Van Thanh) and their adjacent residential areas for comparison. The median size of the parks is 8.9 ha (IQR = 7.7).

Sampling *Aedes* larvae

Aedes mosquito larvae were collected throughout the parks during the period between September and early October 2014, in the middle of the rainy season. Larvae were obtained from potential water holding habitats with pipets, dippers, or aquatic nets. Habitat types were classified into 9 categories: bucket, cup, discarded containers, plant, planter, jar, water tank, flower vase, and other. Habitat location was categorized into indoor, vegetation shade, under eaves, and open outdoor. The larval survey excluded ponds, ditches, puddles, and running water - locations where *A. aegypti* and *A. albopictus* are unlikely to inhabit. When habitats were small enough, field workers tried to capture all larvae using aquatic nets and dippers. For larger habitats, an aquatic net was passed through the water at least 5 times. Collected larvae were preserved in 75% ethanol and identified to species in the laboratory.

In an adjacent residential area, 20 transect lines were established from the boundary of each park using the geographical information system, GIS (ArcGIS ESRI Inc, Redlands, CA, USA). The transect lines were separated from each other by roughly equal distances. For the larval survey the closest house was selected 250 m from the park boundary on arbitrarily selected 1 of 20 lines. The next sampling site was established at 500 m from the park on the next line clockwise. Similarly, two sampling sites were established at distances of 750 m and 1000 m on the next two lines, respectively. This process was repeated with the remaining transect lines, and a total of 80 houses were selected for each adjacent residential area. A total

of 480 houses were targeted for 6 adjacent residential areas. Field workers searched for *Aedes* larvae throughout the indoor and outdoor habitats using the same procedure described for the parks.

Sampling *Aedes* adults

The adult mosquito survey was conducted during the same period as the larval survey. Within each park the areas where visitors cannot access were identified through field observation. In the accessible areas, 48 equally spaced sampling sites were established using GIS. Adult mosquito sampling was conducted at 12 sites between 6:00 and 8:00 on the first day. At each site, 6 equally spaced sampling points were established in an area of 100 m^2. A collector stood at each sampling point, and for 1 min collected approached mosquitoes with a net, followed by a pause of 1 min. The cycle of sampling and pausing was repeated 5 times at each point, for a total of 5 min of sampling. Captured mosquitoes were transferred to a 15 ml plastic centrifuge tube using an aspirator during the interval pauses. The sampling procedure was repeated at the 5 remaining points. Multiple collectors participated in the survey to complete samplings at 12 sites in the morning hours. In the afternoon hours (between 16:00 and 18:00), this process was repeated at 12 different sites, and at 24 remaining sites in the same park on the following day. The adult survey was planned to have a total of 288 samplings for each park and a total of 1,728 samplings within the 6 parks.

In each of 6 adjacent residential areas, 16 lines were drawn from the park boundary using GIS. The lines were systematically assigned 4 different distances in the same manner for the larval survey. At each distance site, the 3 closest houses were identified. Within each house, 3 equally spaced sampling points were established in the yard and in the house, respectively, for a total of 6 sampling points. The survey targeted 4 different distance lines in the morning hours and in the afternoon hours on the first day, respectively. Mosquitoes were sampled at

the remaining 8 lines on the following day. The survey planned to have a total of 288 sampling points with 48 houses in each residential area, and a total of 1,728 points for 6 residential areas.

Statistical analysis

Correspondence analysis with the R package “MASS” was used to explore the relationships between larval habitat types, habitat locations, and distances from the parks [24]. The relationships of these variables with each mosquito species were also examined with correspondence analysis. The occurrence of mosquito species was categorized into “presence of both species”, “*A. aegypti* only”, “*A. albopictus* only”, and “absence of both species." A generalized linear regression model was used to compare the larval occurrences of each species between the habitat locations, between indoor and outdoor habitats, between each distance from the parks and parks, and between the parks and residential areas using the R-package “lme4” and “glmmTMB” [25,26]. Habitat types, geographical areas, habitat locations, and distances were considered as potential covariates. Independence of each covariate was assessed with variation inflation factor, chi-square test, and Pearson product moment correlation or Cramér's V with R package “recompanion” [27]. Collectors, dates, and sampling sites were considered as potential random factors, and a caterpillar plot was used to determine whether these variables should be included in the model. Since log-binomial mixed models with the frequentist approaches often fail to converge, and the outcomes were binary, a prevalence ratio (PR) and confidence interval were estimated with Poisson regression models [28,29].

Adult densities between the parks and residential areas were compared with quasi-Poisson regression models, Poisson regression models, negative binomial models, or zero-inflated models; and collectors, dates, and sampling sites were considered as potential random factors.

An adult density was defined as the number of mosquitoes per sampling for 5 min. The geographical areas and sampling time (morning or afternoon) were considered as potential confounding factors. Since adult mosquitoes were sampled from indoor and outdoor habitats at each house, the densities of each mosquito species were also compared between both habitats.

Results

Occurrence of larvae

The larval survey found 1,988 potential habitats that were holding water. Buckets were most abundant followed by flower vases, cups, and discarded containers (Table 1). A correspondence analysis showed that cups, buckets, vases, and water tanks were associated with the residential areas regardless of distance from the parks, while discarded containers, plants, and planters were associated with the parks (Fig. 1A). Cups, buckets, and vases were associated with indoor habitats; and jars, planters, and water tanks were associated with roof eaves and open outdoor habitats in the residential areas (Fig. 1B). Discarded containers were associated with vegetation shade (Fig. 1B), and most vegetation shade habitats were found in the parks (Table 2, Fig. 1C). The difference in the composition of potential habitat types between the residential areas and parks was statistically significant (Table 3).

Table 1. Relationships of potential water holding habitat types with distance from the parks and the locations. Numbers in parentheses are pro[illegible]ns of each habitat type in each distance site and habitat location.

	Bucket	Cup	Discard	Jar	Plant	Planter	Tank	Vase	Others	Total
Park	23 (3.6)	16 (2.5)	143 (22.2)	45 (7.0)	37 (5.8)	144 (22.4)	47 (7.3)	19 (3.0)	169 (26.3)	643 (100)
Residential	406 (30.2)	246 (18.3)	48 (3.6)	73 (5.4)	2 (0.2)	33 (2.5)	136 (10.1)	250 (18.6)	151 (11.2)	1345 (100)
250 m	80 (18.4)	53 (20.2)	13 (6.8)	26 (22.0)	1 (2.6)	9 (5.1)	33 (18.0)	74 (27.5)	37 (11.6)	326 (100)
500 m	117 (27.3)	86 (32.8)	11 (5.8)	13 (11.0)	1 (2.6)	6 (3.4)	38 (20.8)	59 (22.0)	44 (13.8)	375 (100)
750 m	105 (24.5)	43 (16.4)	8 (4.2)	19 (16.1)	0	12 (6.8)	29 (15.9)	19 (16.1)	21 (6.6)	302 (100)
1000 m	104 (24.2)	64 (24.4)	16 (8.4)	15 (12.7)	0	6 (3.4)	36 (19.7)	15 (12.7)	49 (15.3)	342 (100)
Indoor	332 (34.4)	226 (23.4)	8 (0.8)	35 (3.6)	0 (0)	10 (1.0)	76 (7.9)	207 (21.4)	72 (7.5)	966 (100)
Outdoor	97 (9.5)	36 (3.5)	183 (17.9)	83 (8.1)	39 (3.8)	167 (3.8)	107 (10.5)	62 (6.1)	28 (24.3)	1022 (100)
Under eaves	52 (23.9)	16 (7.4)	26 (11.9)	17 (7.8)	0 (0)	13 (6.0)	23 (10.6)	23 (10.6)	48 (22.0)	218 (100)
Vegetation shade	20 (5.5)	2 (0.6)	102 (28.2)	15 (4.1)	28 (7.7)	74 (20.4)	15 (4.1)	4 (1.1)	102 (28.2)	362 (100)
Other outdoor sites	25 (5.7)	18 (4.1)	55 (12.4)	51 (11.5)	11 (2.5)	80 (18.1)	69 (15.6)	35 (7.9)	98 (22.2)	442 (100)
Total	429 (21.6)	262 (13.2)	191 (9.6)	118 (5.9)	39 (2.0)	177 (8.9)	183 (9.2)	269 (13.5)	320 (16.1)	1988 (100)

Figure 1. Correspondence analysis plots between (A) habitat types and distances from the parks, (B) habitat types and locations, (C) habitat locations and distances, (D) habitat types and mosquitoes, (E) habitat locations and mosquitoes, and (F) distances and mosquitoes.

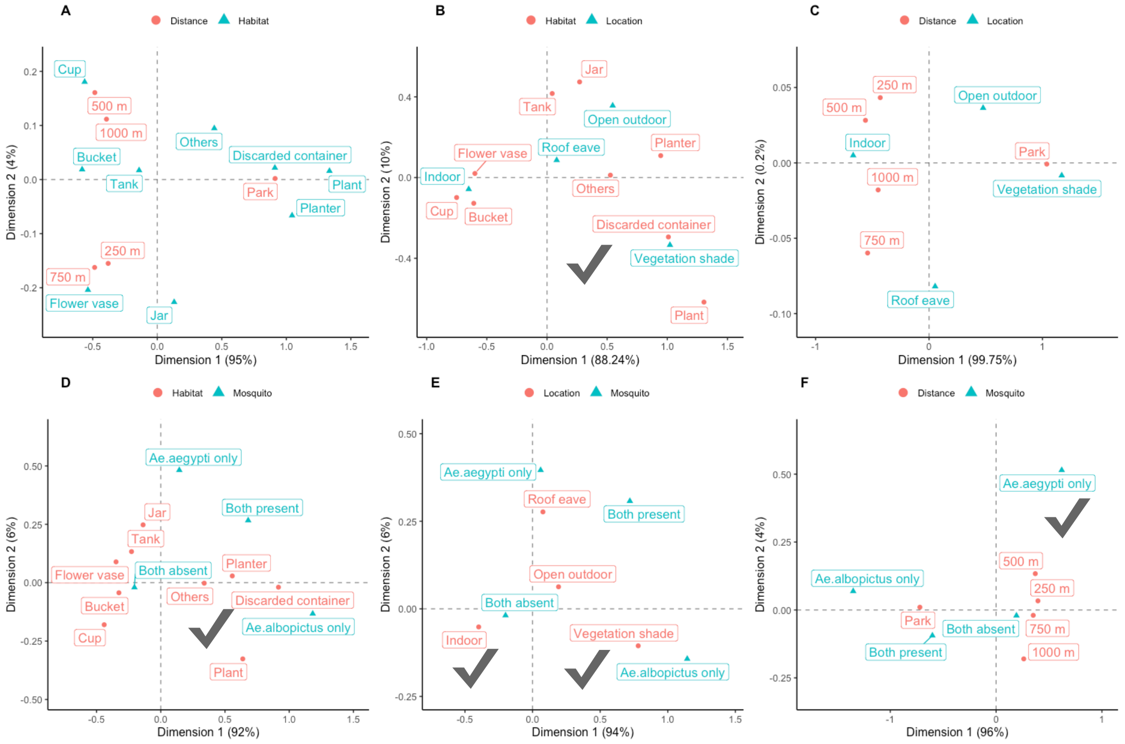

Table 2. Relationships between habitat locations and distances from the parks. Numbers in parentheses are pro[illegible]ons of each habitat type within each distance and habitat location.

	Indoor	Under eaves	Vegetation shade	Open outdoor sites	All outdoor sites	Total
Park	11 (1.7)	76 (11.8)	315 (49.0)	241 (37.5)	632 (98.3)	643 (100)
Residential	955 (71.0)	142 (10.6)	47 (3.5)	201 (14.9)	393 (29.0)	1348 (100)
250 m	221 (67.8)	32 (14.7)	15 (4.6)	58 (17.8)	105 (32.2)	326 (100)
500 m	280 (74.7)	35 (9.3)	8 (2.1)	52 (13.9)	95 (25.3)	375 (100)
750 m	219 (72.5)	37 (12.3)	7 (2.3)	39 (12.9)	83 (27,5)	302 (100)
1000 m	235 (68.7)	38 (11.1)	17 (5.0)	52 (15.2)	107 (31.3)	342 (100)
Total	966 (48.6)	218 (11.0)	362 (18.2)	442 (22.2)	1022 (51.4)	1988 (100)

Table 3. Relationships between the covariates indicated by chi-square tests and Camér V.

	Chi-square	df	p-value	Cramér V
Residential/Park versus geographical area	102	5	< 0.001	0.23
Residential/Park versus distance	1988	4	< 0.001	1
Residential/Park versus habitat	792	8	< 0.001	0.63
Residential/Park versus habitat location	1024	3	< 0.001	0.71
Geographical area versus distance	175	20	< 0.001	0.16
Geographical area versus habitat	237	40	< 0.001	0.16
Geographical area versus habitat location	145	15	< 0.001	0.16
Distance versus habitat	837	32	< 0.001	0.33
Distance versus habitat location	1031	12	< 0.001	0.42
Habitat versus habitat location	1043	24	< 0.001	0.42

Aedes larvae were found at 295 (20%) of 1,988 habitats. Discarded containers and planters composed 60% of the positive habitats. Despite large numbers of buckets and cups, the occurrence of *Aedes* larvae were significantly lower compared with the other habitats while their presence was significantly higher in discarded containers (Table 4). At a species level, *A. aegypti* and *A. albopictus* were found at 9% and 16% of the potential habitats, respectively. A correspondence analysis revealed that both species were positively associated with discarded containers and planters, and *A. albopictus* was negatively associated with flower vases, buckets, water tanks, jars, and cups (Fig. 1D). The occurrences of both species were significantly lower in cups. While the occurrence of *A. albopictus* was also significantly lower in water tanks, it was significantly higher in discarded containers. The logistic regression analysis for each habitat type was adjusted for the variables of distance, habitat location, and geographical area; but the variable of residential/park was excluded because of

the high association with habitat types (Table 3). The optimal models included only sampling site as a random intercept.

Table 4. Occurrence (%) and prevalence ratio (95% C[illegible]ach mosquito species in each habitat type. The ratio was estimated based on the other habitats as a reference, and an asterisk shows statistical significance with a p-value of 0.05.

	Bucket	Ref	Cup	Ref	Discard	Ref	Plant	Ref	Planter	Ref	Jar	Ref	Tank	Ref	Vase	Ref	Other	Ref	Total
Ae. aegypti																			
Positive	20 (4.7)	156 (10.0)	0 (0)	176 (10.2)	33 (17.3)	143 (9.0)	2 (5.1)	174 (8.9)	28 (15.8)	148 (8.2)	16 (13.6)	160 (8.6)	19 (10.4)	157 (8.7)	20 (7.4)	156 (9.1)	38 (11.9)	138 (8.3)	176 (8.9)
Negative	409 (95.3)	1403 (90.0)	262 (100)	1550 (89.8)	158 (82.7)	1654 (92.0)	37 (94.9)	1775 (92.3)	149 (84.2)	1663 (91.8)	102 (86.4)	1710 (91.4)	164 (89.6)	1648 (91.3)	249 (92.6)	1563 (90.9)	282 (88.1)	1530 (91.7)	1812 (91.4)
Unadjusted PR	0.46* 0.28-0.78	1	0 0	1	1.93* 1.25-2.97	1	0.60 0.14-2.56	1	1.91* 1.19-3.09	1	1.51 0.86-2.68	1	1.07 0.64-1.79	1 ref	0.93 0.56-1.55	1	1.31 0.88-1.96	1	-
Adjusted PR	0.61 0.37-1.00	1	0* 0	1	1.51 0.98-2.32	1	0.50 0.12-2.15	1	1.49 0.93-2.39	1	1.37 0.78-2.38	1	0.95 0.57-1.57	1 ref	1.36 0.81-2.29	1	1.00 0.67-1.48	1	-
Ae. albopictus																			
Positive	20 (4.7)	301 (19.3)	1 (0.4)	320 (18.5)	93 (48.7)	228 (12.7)	14 (35.9)	307 (15.4)	66 (37.3)	255 (14.1)	13 (11.0)	308 (16.5)	16 (8.7)	305 (16.9)	9 (3.3)	312 (18.2)	89 (27.8)	232 (13.9)	321 (16.1)
Negative	409 95.3)	1258 (80.7)	261 (99.4)	1406 (81.5)	98 (51.3)	1569 (87.3)	25 (64.1)	1642 (84.2)	111 (62.7)	1556 (85.9)	105 (89.0)	1562 (83.5)	167 (91.3)	1500 (83.1)	260 (96.7)	1407 (81.9)	231 (72.2)	1436 (86.1)	1667 (83.9)
Unadjusted PR	0.33* 0.20-0.54	1	0.03* 0-0.26	1 (	2.73* 1.95-3.80	1	2.26* 1.04-4.90	1	1.90* 1.26-2.86	1	0.63 0.34-1.18	1	0.55* 0.32-0.95	1 ref	0.31* 0.15-0.63	1	1.65* 1.19-2.28	1	-
Adjusted PR	0.86 0.52-1.40	1	0.08* 0.01-0.59	1	1.74* 1.32-2.29	1	0.93 0.51-1.70	1	1.01 0.73-1.39	1	0.64 0.35-1.16	1	0.56* 0.33-0.94	1 ref	0.75 0.37-1.52	1	1,10 0.84-1.44	1	-
Aedes																			
Positive	28 (6.5)	367 (23.5)	40 (10.7)	394 (22.8)	102 (53.9)	292 (16.5)	15 (38.5)	380 (19.5)	75 (42.4)	320 (17.7)	23 (19.5)	372 (19.9)	24 (13.1)	371 (20.6)	23 (8.6)	372 (21.6)	103 (32.2)	292 (17.5)	295 (19.9)
Negative	401 (93.5)	1192 (76.5)	261 (99.6)	1332 (77.2)	83 (46.1)	1505 (83.8)	24 (61.5)	1569 (80.5)	102 (57.6)	1491 (82.3)	95 (80.5)	1498 (80.1)	159 (86.9)	1434 (90.0)	246 (91.5)	1347 (78.4)	217 (67.8)	1376 (82.5)	1593 (80.1)
Unadjusted PR	0.32* 0.21-0.48	1	0.02* 0-0.17	1	2.64* 1.98-3.52	1	2.04* 1.03-4.06	1	1.82* 1.29-2.59	1	0.94 0.59-1.51	1 ref	0.63* 0.40-0.99	1	0.54* 0.34-0.88	1	1.65* 1.25-2.18	1	-
Adjusted PR	0.65* 0.43-0.98	1	0.05* 0 - 0.35	1	1.66* 1.28-2.14	1	0.96 0.54-1.70	1	1.07 0.80-1.44	1	0.91 0.58-1.43	1 ref	0.66 0.42-1.01	1	1.15 0.72-1.83	1	1.12 0.87-1.44	1	-

While the occurrence of *A. aegypti* was positively associated with indoor habitat, roof eaves, and open outdoor habitats, *A. albopictus* was positively associated with vegetation shade (Fig. 1E). The occurrence was significantly higher with all outdoor habitats for both species compared with indoor habitats (Table 5). The variable of residential/park was excluded from these regression models because its association was high with habitat locations (Table 3). The optimal models included only sampling site as a random intercept.

Table 5. Occurrence (%) and prevalence ratio (95% CI) of each mosquito species collected at four habitat locations. The ratio was estimated based on indoor habitat, and an asterisk shows statistical significance with a p-value of 0.05.

	Indoor	Outdoor			
	Indoor	Under eaves	Vegetation shade	Open outdoor	All outdoor
Ae. aegypti					
Positive	34 (3.5)	37 (17.0)	50 (13.8)	55 (12.4)	142 (13.9)
Negative	932 (96.5)	181 (83.0)	312 (86.2)	387 (87.6)	880 (86.1)
Unadjusted PR	1 (ref)	4.80 (2.89 - 7.99)*	3.62 (2.25 - 5.82)*	3.44 (2.17 - 5.44)*	3.79 (2.54 - 5.65)*
Adjusted PR	1 (ref)	3.79 (2.23 - 6.40)*	2.89 (1.60-5.24)*	2.54 (1.52 - 4.26)*	3.03 (1.90 - 4.85)*
Ae. albopictus					
Positive	16 (1.7)	43 (19.7)	159 (43.9)	103 (23.3)	305 (29.8)
Negative	950 (98.4)	175 (80.3)	203 (56.1)	339 (76.7)	717 (70.2)
Unadjusted PR	1 (ref)	11.93 (6.56 - 21.67)*	22.94 (13.39 - 39.33)*	12.36 (7.15 - 21.38)*	15.44 (9.15 - 26.05)*
Adjusted PR	1 (ref)	4.22 (2.20 - 8.08)*	4.07 (2.11 - 7.88)*	3.45 (1.81 - 6.56)*	3.83 (2.07 - 7.11)*
Aedes spp.					
Positive	38 (3.9)	60 (27.5)	174 (48.1)	123 (27.8)	357 (34.9)
Negative	928 (96.1)	158 (72.5)	188 (51.9)	319 (72.2)	665 (65.1)
Unadjusted PR	1 (ref)	7.5 (4.60 - 10.81)*	11.10 (7.64 - 16.14)*	6.44 (4.39 - 9.45)*	8.06 (5.66 - 11.46)*
Adjusted PR	1 (ref)	3.66 (2.30 - 5.80)*	3.42 (2.12 - 5.52)*	2.72 (1.73 - 4.29)*	3.16 (2.06 - 4.84)*
No of habitats	966	218	362	442	1022

Nearly 70% of the *Aedes* larval positive sites were found in the parks. The proportions of habitats containing *Aedes* larvae were 44% and 9% for the parks and residential areas, respectively. Logistic regression analyses revealed that the proportion was significantly higher in the parks for both species (Table 6). However, a correspondence analysis indicated that *A. albopictus* was positively associated with the parks while *A. aegypti* was positively associated with the residential areas (Fig. 1F). Although the relationships of *A. albopictus* occurrence were not clear with the distances from the parks (Fig. 1F), the occurrence of *A. aegypti* was not significantly lower at the distances of 500 m and 750 m compared with the parks (Table 6). In particular, the PR at 500 m was as high as that of the parks. Nevertheless, the reduction in proportion of positive sites was similar among all distances when both species were combined. In the regression models for comparing parks and residential areas, the covariates included were only residential/park and geographical area, because the associations of residential/park were high with the other variables (Table 3). The optimal models also included sampling site as a random intercept, but not other variables.

Table 6. Occurrence (%) and prevalence ratio (95% CI) of each mosquito species at four distances from the parks. The ratio was estimated based on the mosquito populations of the parks and an asterisk shows statistical significance with a p-value of 0.05.

	Park	Residential					Total
	0 m	250 m	500 m	750 m	1000 m	All residential	
Ae. aegypti							
Positive	73 (11.4)	19 (5.8)	38 (10.1)	23 (7.6)	23 (6.7)	103 (7.7)	176 (8.9)
Negative	570 (88.6)	307 (94.2)	337 (89.9)	279 (92.4)	319 (93.3)	1242 (92.3)	1812 (91.1)
Unadjusted PR	1 (ref)	0.52 (0.28-0.95)*	0.95 (0.58-1.58)	0.73 (0.41-1.29)	0.57 (0.3-1.01)	0.69 (0.47-1.01)	-
Adjusted PR	1 (ref)	0.51 (0.28-0.94)*	0.95 (0.58-1.55)	0.63 (0.36-1.11)	0.54 (0.30-0.95)*	0.66 (0.45-0.96)*	-
Ae. albopictus							
Positive	265 (41.2)	5 (1.5)	15 (4.0)	12 (4.0)	24 (7.0)	56 (4.2)	321 (16.1)
Negative	378 (58.8)	321 (98.5)	360 (96.0)	290 (96.0)	318 (93.0)	1289 (95.8)	1667 (83.9)
Unadjusted PR	1 (ref)	0.04 (0.02-0.10)*	0.10 (0.06-0.17)*	0.10 (0.05-0.18)*	0.18 (0.11-0.28)*	0.10 (0.07-0.14)*	-
Adjusted PR	1 (ref)	0.04 (0.02-0.10)*	0.10 (0.06-0.17)*	0.09 (0.05-0.16)*	0.17 (0.11-0.27)*	0.10 (0.07-0.14)*	-
Aedes spp.							
Positive	279 (43.4)	22 (6.7)	40 (10.7)	27 (8.9)	27 (7.9)	116 (8.6)	395 (19.9)
Negative	364 (56.6)	304 (93.3)	335 (89.3)	275 (91.1)	315 (92.1)	1229 (91.4)	1593 (80.1)
Unadjusted PR	1 (ref)	0.16 (0.10-0.26)*	0.26 (0.18-0.38)*	0.22 (0.14-0.34)*	0.19 (0.12-0.29)*	0.21 (0.16-0.17)*	-
Adjusted PR	1 (ref)	0.16 (0.10-0.26)*	0.26 (0.16-0.38)*	0.20 (0.13-0.31)*	0.18 (0.12-0.28)*	0.20 (0.15-0.26)*	-
No of habitats	643	326	375	302	342	1345	1988

Adult mosquito abundance

Adult mosquito sampling was conducted at 2,640 (76%) of the 3,456 initially planned sampling points, and the missed sites were largely due to bad weather conditions. Of the sampled sites, 24 were excluded from the analyses because of incomplete datasets. For the resulting 2,616 points, 1,584 (61%) and 1,032 (39%) were in the parks and residential areas, respectively (Table 7). A total of 1,117 *Aedes* mosquitoes were collected, 185 (17%) of which were *A. aegypti* and 932 (83%) were *A. albopictus*.

Figure 2. Densities of adult mosquitoes collected in the parks and at residential sites with four different distances from the parks, residential indoor and outdoor sites, during the two time periods and six geographical areas.

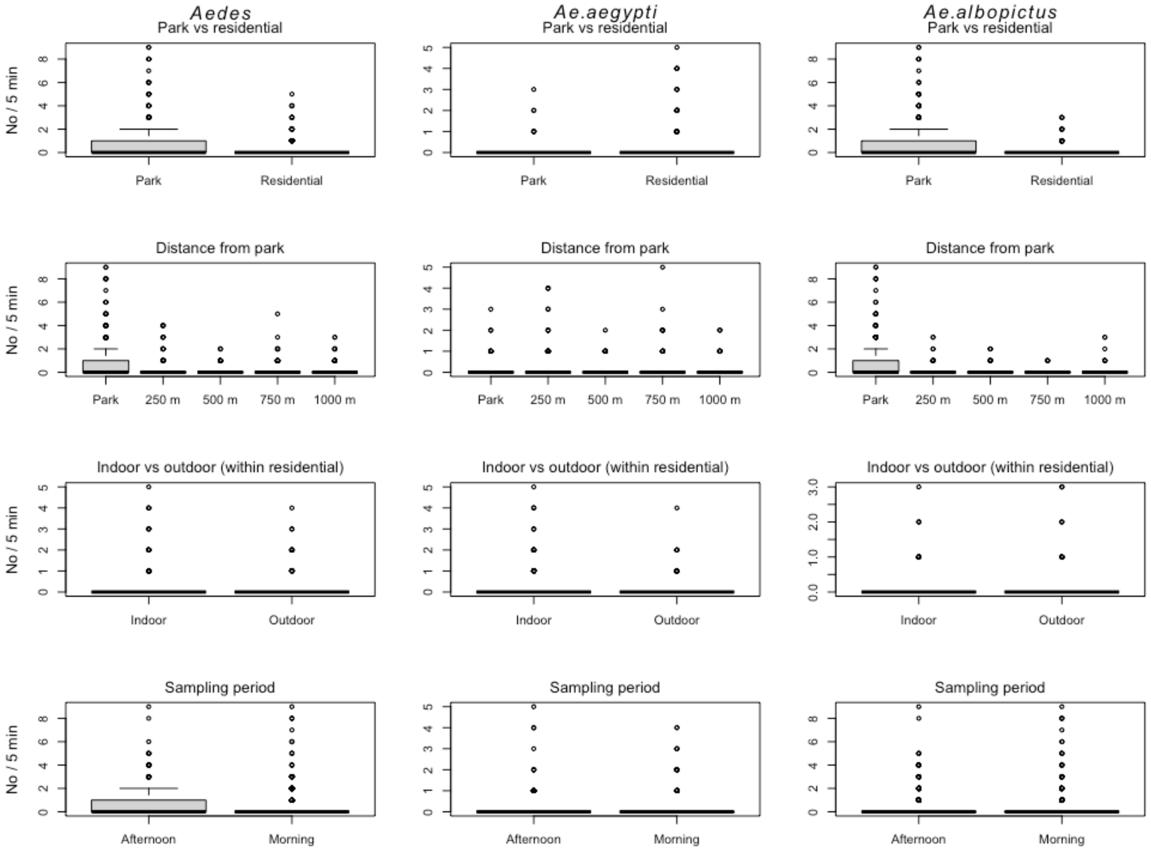

Table 7. Comparisons of adult mosquito abundance between the parks and residential areas, between the parks and different distances from the parks, between the indoor and outdoor sites, and between the morning and afternoon hours. Prevalence ratios (95% CI) at each distance site were estimated based on the mosquito density in the parks, the PRs of the outdoor sites were based on the indoor sites, and the PRs of the afternoon hours were based on the morning hours. An asterisk shows statistical significance with a p-value of 0.05.

	Park	Residential							Residential and park	
	0 m	250 m	500 m	750 m	1000 m	All	Indoor	Outdoor	Morning	Afternoon
Ae. aegypti										
Unadjusted PR (95% CI)	1	15.63* (7.49-32.61)	8.23* (3.54-19.10)	11.70* (5.52-24.82)	9.11* (4.22-19.69)	11.31* (6.25-20.46)	1 (ref)	0.46* (0.33-0.65)	1 (ref)	0.90 (0.52-1.56)
Adjusted PR (95% CI)	1	15.12* (7.29-31.37)	8.29* (3.60-19.08)	11.90* (5.65-25.06)	9.36* (4.35-20.12)	11.35* (6.29-20.50)	1 (ref)	0.47* (0.30-0.73)	1 (ref)	0.82 (0.49-1.37)
Ae. albopictus										
Unadjusted PR (95% CI)	1	0.13* (0.07-0.25)	0.15* (0.08-0.30)	0.04* (0.02-0.11)	0.08* (0.04-0.16)	0.10* (0.06-0.14)	1 (ref)	0.97 (0.59-1.60)	1 (ref)	1.20 (0.84-1.70)
Adjusted PR (95% CI)	1	0.14* (0.08-0.26)	0.16* (0.08-0.30)	0.04* (0.02-0.11)	0.08* (0.04-0.17)	0.10* (0.07-0.15)	1 (ref)	1.03 (0.62-1.72)	1 (ref)	1.38* (1.04-1.84)
Aedes spp.										
Unadjusted PR (95% CI)	1	0.57* (0.37-0.87)	0.38* (0.23-0.63)	0.34* (0.21-0.54)	0.30* (0.18-0.48)	0.39* (0.30-0.50)	1 (ref)	0.57* (0.42-0.76)	1 (ref)	1.14 (0.87-1.49)
Adjusted PR (95% CI)	1	0.58* (0.39-0.88)	0.38* (0.23-0.63)	0.35* (0.22-0.56)	0.31* (0.19-0.49)	0.40* (0.31-0.52)	1 (ref)	0.58* (0.43-0.77)	1 (ref)	1.24 (0.98-1.59)
No of sampling points	1,584	270	216	270	276	1,032	528	528	1,475	1,141

The numbers of mosquitoes collected in the parks and the residential areas were 897 (80%)

and 220 (20%), respectively (Fig. 2). The numbers of *A. aegypti* and *A. albopictus* were 24 (3%) and 873 (97%) in the parks, and 161 (73%) and 61 (27%) in the residential areas, respectively. The density of *A. aegypti* was significantly greater in the residential areas, and it was more than ten-fold (Table 7). The density of *Ae. aegypti* was greater at all distances compared with the parks, and the density was greatest at 250 m (Fig. 2). The increases were statistically significant at all distances from the parks. In contrast, the density of *Ae. albopictus* was significantly higher in the parks. The adjusted PRs indicate that the reductions were around 90% in the residential areas, and the reductions were greater at 750 m and 1000 m. Overall, the densities of *Aedes* mosquitoes in the parks were significantly greater compared with the residential areas, and reduced with an increase of distance from the parks. The adult data were analyzed with quasi-Poisson regression models that incorporated sampling site as a random factor. The variables of geographical area and sampling period were included as confounding factors.

Quasi-Poisson models revealed that the density of *A. aegypti* in the residential areas was significantly less in the outdoor habitat compared with the indoor habitat. However, the difference in density between the habitats was not statistically significant for *A. albopictus* (Table 7). When the two species were combined, the density was significantly lower in the outdoor habitats in the residential areas. The optimal regression models also included geographical area and sampling period as confounding factors and sampling site as a random factor.

Between the morning and afternoon sampling hours, the difference in density was not statistically significant for *A. aegypti*; however, the density of *A. albopictus* was significantly greater in the afternoon (Table 7). When the two species were combined, the difference in density was not statistically significant. The optimal quasi-Poisson models included

geographical area and park/residential as confounding factors and sampling site as a random factor.

Discussion

The present study found that the densities of adult *Aedes* mosquitoes, in particular *A. albopictus*, were greater in the major parks in Ho Chi Minh City compared with the adjacent residential areas. While nearly all *Aedes* mosquitoes collected in the parks were *A. albopictus, Ae. aegypti* was predominant in the adjacent residential areas. Past studies reported a positive association of *A. albopictus* with vegetation in rural areas [15,16,17]. The present study found a similar phenomenon within an urban area at a smaller spatial scale. The study in São Paulo also found a positive association of *A. albopictus* with vegetation, in a much larger park [7]. The vegetation in the parks of Ho Chi Minh City is apparently sparser than in São Paulo, but is sufficient to provide a suitable environment for this mosquito species.

Most breeding habitats in the parks were not found directly on plants; rather, in artificial habitats such as discarded containers and planters in vegetation shade. The results still suggest that shade created by abundant vegetation in the parks provides a suitable environment for *A. albopictus.* Tree shade may create a microclimate suitable for this mosquito species, and nutrients from leaf litter may be an energy source and enhance the development of larvae [26]. *Aedes albopictus* adults are known to ingest sugars from understory vegetation [30,31], and they may seek blood meals more actively in the shade created by trees [32].

For both species the prevalence of positive larval habitats was lower in the residential areas than the parks, suggesting that the residential environment is less suitable regardless of the taxa. One plausible explanation is that the residential habitats are unstable. Residents

physically remove potential habitats more often and replace or empty the water in the habitats; and as a result the habitat lifespans in the residential areas become shorter. Vector control activities may also explain the lower prevalence of larvae, and the lower density of adults in the residential areas. The local government often conducts outdoor insecticide spraying during a dengue outbreak; however, this was not the case before and during the present study in the 2014 season. Residents may still use insecticides for personal protection, but the effects of individual effort on mosquitoes may not be extensive.

The greater density of *Aedes* mosquitoes implies that infection risk is considerable in the parks. The viral transmission dynamics is not only affected by vector population size, but also by human population density and movement [33,34]. Many people visit the parks during the day when vectors actively seek blood meals, and the temporal increase in traffic increases human-vector contact in the parks. The present study found that the density of *A. albopictus* was higher in the afternoon. The result suggests that this mosquito species seeks blood meals more actively in the afternoon when more people visit and stay in the parks.

Visitors of the large urban parks come from various parts from within, as well as outside the city. The extensive human movements increase the chance of virus introduction to the vector populations in the parks. Visitors newly infected by the vectors in the parks may spread the viruses to other locations within and outside the city [35]. The movements of people and viruses would be less within the residential areas compared with the parks, unless large commercial activities are present; and on the whole viruses would be transmitted more efficiently and effectively in the urban parks than the residential areas.

The present study found that the density of *A. aegypti* was higher in the residential areas than in the parks; and in particular the density was greater inside houses. This mosquito species is

considered to be the most efficient vector of DENV because of its greater anthropophagy and virus susceptibility [36,37]. However, the vectorial capacity varies by DENV strain, vector population, and climate [38–41]. *Aedes albopictus* has an opportunistic host seeking behavior and may become more anthropophilic in the urban parks because of the absence of large animals and the concentration of humans [42].

The density of *A. aegypti* was greatest at 250 m distance from the parks, and the density of *A. albopictus* was higher at 250 m and 500 m compared with the other distances. These phenomena may be explained by a spillover of mosquitoes from the parks to the close residential areas. Otherwise, mosquitoes may be concentrated in the area to seek blood meals from both residents and park visitors. While the former case may be applied to *A. albopictus* which is abundant in the parks, the latter case may be applied to *A. aegypti* which is predominant in the residential areas. It is known that the oviposition of *A. aegypti* is positively associated with the peripheral area of the urban park in São Paulo [7]. Such boundary or edge effects are also observed in a cluster randomized control study of mosquito nets against anopheline mosquitoes in Africa. The density of anopheline mosquitoes was also reduced in the neighboring village where bed nets were not distributed [43].

The abundance of *A. albopictus* in the parks may extend the transmission season. As the dry season progresses, the abundance of *A. albopictus* increases relative to *A. aegypti* [44], because the latter species produces superior desiccation-resistant eggs to survive the dry season and accordingly reduces the number of adults [45,46]. Vegetation in the parks may retain humidity and provide nutrients for *A. albopictus* during the dry season. As a result, viral transmission may persist in the parks during the dry season via the *A. albopictus* population as a reservoir of viruses. As survived eggs of *A. aegypti* hatch in the early rainy season, the viruses may spread rapidly from the parks to the residential areas with an increase

of *A. aegypti* adults [45,46]. Moreover, the abundance of *A. albopictus* in the parks may increase the risk of other viral diseases such as chikungunya and zika in the urban area. Reportedly, *A. albopictus* has a greater susceptibility to chikungunya virus than *A. aegypti* [47,48]. An evidence of chikungunya virus infection has been reported from Ho Chi Minh City [49], and most Zika cases in the 2016-2017 outbreak in Vietnam were reported from the city [50].

Limitation

The present study was conducted cross-sectionally in the middle of the rainy season, and temporally and spatially only covered a small portion of the vector population. Therefore, the information from the preset study is too little to understand the entire vector population dynamics in the parks and the adjacent residential areas. Although a large number of larvae were sampled systematically, the data from the present study were qualitative. Because the sampling methods differed according to types and sizes of larval habitat, it is not easy to standardize larval densities. Although the potential risk is inferred based on the abundance of vectors, the risk must be directly assessed with the prevalence of human infections.

Implication

The role of urban parks in viral transmission should not be underestimated, and urban parks should be included in current vector control programs. Other public places, such as schoolgrounds, should also be given attention. As suggested by past studies [6–9], the present study confirms the importance of artificial vessels such as discarded containers for breeding mosquitoes in the urban parks. The results from the present study suggest that periodic removal of artificial habitats will reduce vectors and lessen the infection risk in urban areas.

Acknowledgments

The first author (TH) is grateful for the academic support from the Program for Nurturing Global Leaders in Tropical and Emerging Communicable Diseases, Graduate School of Biomedical Sciences, Nagasaki University. We thank the staff of Pasteur Institute in Ho Chi Minh city (PIHCMC) for laboratory and field assistance as well as the staff from Preventive Medicine Center in Ho Chi Minh city (now named as HCDC) and from districts and communes for field assistance. In addition, we thank Mark L. Wilson, Takashi Tsunoda and Ataru Tsuzuki for constructive comments.This research was supported by the Japan Agency for Medical Research and Development (AMED) under Grant Number JP21wm0125006. The funders had no role in study design, data collection and analysis, decision to publish, or preparation of the manuscript.

References

1. World Health Organization. Dengue and severe dengue. [cited 29 May 2021]. Available: https://www.who.int/news-room/fact-sheets/detail/dengue-and-severe-dengue

2. Bhatt S, Gething PW, Brady OJ, Messina JP, Farlow AW, Moyes CL, et al. The global distribution and burden of dengue. Nature. 2013;496: 504–507. doi:10.1038/nature12060

3. Guo C, Zhou Z, Wen Z, Liu Y, Zeng C, Xiao D, et al. Global Epidemiology of Dengue Outbreaks in 1990–2015: A Systematic Review and Meta-Analysis. Front Cell Infect Microbiol. 2017;7: 317. doi:10.3389/fcimb.2017.00317

4. Azami NAM, Moi ML, Salleh SA, Neoh H, Kamaruddin MA, Jalal NA, et al. Dengue epidemic in Malaysia: urban versus rural comparison of dengue immunoglobulin G seroprevalence among Malaysian adults aged 35–74 years. Trans R Soc Trop Med Hyg. 2020;114: 798–811. doi:10.1093/trstmh/traa056

5. Doum D, Overgaard HJ, Mayxay M, Suttiprapa S, Saichua P, Ekalaksananan T, et al. Dengue Seroprevalence and Seroconversion in Urban and Rural Populations in Northeastern Thailand and Southern Laos. IJERPH. 2020;17: 9134. doi:10.3390/ijerph17239134

6. Dowling Z, Ladeau SL, Armbruster P, Biehler D, Leisnham PT. Socioeconomic Status Affects Mosquito (Diptera: Culicidae) Larval Habitat Type Availability and Infestation Level. J Med Entomol. 2013;50: 764–772. doi:10.1603/ME12250

7. García-Rejón JE, López-Uribe MP, Loroño-Pino MA, Farfán-Ale JA, Najera-Vazquez MDR, Lozano-Fuentes S, et al. Productive Container Types for *Aedes aegypti* Immatures in Mérida, México. J Med Entomol. 2011;48: 644–650.

doi:10.1603/ME10253

8. Walker KR, Williamson D, Carrière Y, Reyes-Castro PA, Haenchen S, Hayden MH, et al. Socioeconomic and human behavioral factors associated with *Aedes aegypti* (Diptera: Culicidae) Immature Habitat in Tucson, AZ. J Med Entomol. 2018;55: 955–963. doi:10.1093/jme/tjy011

9. Paul KK, Dhar-Chowdhury P, Haque CE, Al-Amin HM, Goswami DR, Kafi MAH, et al. Risk factors for the presence of dengue vector mosquitoes, and determinants of their prevalence and larval site selection in Dhaka, Bangladesh. PLoS One. 2018;13: e0199457. doi:10.1371/journal.pone.0199457

10. Rajarethinam J, Ong J, Neo Z-W, Ng L-C, Aik J. Distribution and seasonal fluctuations of *Ae. aegypti* and *Ae. albopictus* larval and pupae in residential areas in an urban landscape. Bonizzoni M, editor. PLoS Negl Trop Dis. 2020;14: e0008209. doi:10.1371/journal.pntd.0008209

11. Medeiros-Sousa AR, Ceretti-Júnior W, de Carvalho GC, Nardi MS, Araujo AB, Vendrami DP, et al. Diversity and abundance of mosquitoes (Diptera:Culicidae) in an urban park: Larval habitats and temporal variation. Acta Tropica. 2015;150: 200–209. doi:10.1016/j.actatropica.2015.08.002

12. Kutsuna S, Kato Y, Moi ML, Kotaki A, Ota M, Shinohara K, et al. Autochthonous Dengue Fever, Tokyo, Japan, 2014. Emerg Infect Dis. 2015;21: 517–520. doi:10.3201/eid2103.141662

13. Tsuda Y, Maekawa Y, Ogawa K, Itokawa K, Komagata O, Sasaki T, et al. Biting Density and Distribution of *Aedes albopictus* during the September 2014 Outbreak of Dengue Fever in Yoyogi Park and the Vicinity of Tokyo Metropolis, Japan. Jpn J Infect Dis.

2016;69: 1–5. doi:10.7883/yoken.JJID.2014.576

14. Kobayashi D, Murota K, Fujita R, Itokawa K, Kotaki A, Moi ML, et al. Dengue Virus Infection in Aedes albopictus during the 2014 Autochthonous Dengue Outbreak in Tokyo Metropolis, Japan. Am J Trop Med Hyg. 2018;98: 1460–1468. doi:10.4269/ajtmh.17-0954

15. Mogi M, Khamboonruang C, Choochote W, Suwanpanit P. Ovitrap surveys of dengue vector mosquitoes in Chiang Mai, northern Thailand: seasonal shifts in relative abundance of *Aedes albopictus* and *Ae. aegypti*. Med Vet Entomol. 1988;2: 319–324. doi:10.1111/j.1365-2915.1988.tb00203.x

16. Tsuda Y, Suwonkerd W, Chawprom S, Prajakwong S, Takagi M. Different spatial distribution of *Aedes aegypti* and *Aedes albopictus* along an urban-rural gradient and the relating environmental factors examined in three villages in northern Thailand. J Am Mosq Control Assoc. 2006;22: 222–228. doi:10.2987/8756-971X(2006)22[222:DSDOAA]2.0.CO;2

17. Honório NA, Castro MG, Barros FSM de, Magalhães M de AFM, Sabroza PC. The spatial distribution of *Aedes aegypti* and *Aedes albopictus* in a transition zone, Rio de Janeiro, Brazil. Cad Saude Publica. 2009;25: 1203–1214. doi:10.1590/s0102-311x2009000600003

18. Saifur RGM, Hassan AA, Dieng H, Ahmad H, Salmah MRC, Satho T, et al. Update on temporal and spatial abundance of dengue vectors in Penang, Malaysia. J Am Mosq Control Assoc. 2012;28: 84–92. doi:10.2987/11-6220R.1

19. Lee C, Vythilingam I, Chong C-S, Abdul Razak MA, Tan C-H, Liew C, et al. Gravitraps for Management of Dengue Clusters in Singapore. Am J Trop Med Hyg. 2013;88: 888–

892. doi:10.4269/ajtmh.12-0329

20. Higa Y, Yen NT, Kawada H, Son TH, Hoa NT, Takagi M. Geographic distribution of *Aedes aegypti* and *Aedes albopictus* collected from used tires in Vietnam. J Am Mosq Control Assoc. 2010;26: 1–9. doi:10.2987/09-5945.1

21. Kawada H, Oo SZM, Thaung S, Kawashima E, Maung YNM, Thu HM, et al. Co-occurrence of point mutations in the voltage-gated sodium channel of pyrethroid-resistant *Aedes aegypti* populations in Myanmar. PLoS Negl Trop Dis. 2014;8: e3032. doi:10.1371/journal.pntd.0003032

22. Thu TNT, Dang TN, Duong TTM, Phung ND, Le NH, Tran HTA, et al. A scoring scheme prediction model for dengue outbreaks using weather factors in Ho Chi Minh city, Vietnam. Med Pharm Res. 2021;5: 30–37. doi:10.32895/UMP.MPR.5.1.6

23. Ngwe Tun MM, Nguyen TTT, Ando T, Dumre SP, Soe AM, Buerano CC, et al. Clinical, virological, and cytokine profiles of children infected with dengue virus during the outbreak in Southern Vietnam in 2017. Am J Trop Med Hyg. 2020;102: 1217–1225. doi:10.4269/ajtmh.19-0607

24. Venables B, Ripley B. Modern applied atatistics with S. Springer. 2002. doi:10.1007/b97626

25. Bates D, Mächler M, Bolker B, Walker S. Fitting linear mixed-effects models using lme4. J Stat Soft. 2015;67: 1–48. doi:10.18637/jss.v067.i01

26. Modeling zero-inflated count data with glmmTMB | bioRxiv. [cited 13 Jun 2021]. Available: https://www.biorxiv.org/content/10.1101/132753v1

27. R Companion: R Tutorials. [cited 13 Jun 2021]. Available:

https://rcompanion.org/rcompanion/a_09.html

28. Barros AJ, Hirakata VN. Alternatives for logistic regression in cross-sectional studies: an empirical comparison of models that directly estimate the prevalence ratio. BMC Med Res Methodol. 2003;3: 21. doi:10.1186/1471-2288-3-21

29. Deddens JA, Petersen MR. Approaches for estimating prevalence ratios. Occupational and Environmental Medicine. 2008;65: 501–506. doi:10.1136/oem.2007.034777

30. Braks M a. H, Juliano SA, Lounibos LP. Superior reproductive success on human blood without sugar is not limited to highly anthropophilic mosquito species. Med Vet Entomol. 2006;20: 53–59. doi:10.1111/j.1365-2915.2006.00612.x

31. Müller GC, Xue R-D, Schlein Y. Differential attraction of *Aedes albopictus* in the field to flowers, fruits and honeydew. Acta Trop. 2011;118: 45–49. doi:10.1016/j.actatropica.2011.01.009

32. Sunahara T. Extremely High biting densities of *Aedes albopictus* (Skuse) (Diptera: Culicidae) at a university campus in Nagasaki, Japan. Jpn J Infect Dis. 2019;72: 368–373. doi:10.7883/yoken.JJID.2019.047

33. Chen WJ, Wei HL, Hsu EL, Chen ER. Vector competence of *Aedes albopictus* and *Ae. aegypti* (Diptera: Culicidae) to dengue 1 virus on Taiwan: development of the virus in orally and parenterally infected mosquitoes. J Med Entomol. 1993;30: 524–530. doi:10.1093/jmedent/30.3.524

34. Romeo-Aznar V, Paul R, Telle O, Pascual M. Mosquito-borne transmission in urban landscapes: the missing link between vector abundance and human density. Proc R Soc B. 2018;285: 20180826. doi:10.1098/rspb.2018.0826

35. Kojima G. Autochthonous dengue fever imported to England from Japan, 2014. Emerg Infect Dis. 2015;21: 182–184. doi:10.3201/eid2101.141581

36. Scott TW, Chow E, Strickman D, Kittayapong P, Wirtz RA, Lorenz LH, et al. Blood-feeding patterns of *Aedes aegypti* (Diptera: Culicidae) collected in a rural Thai village. J Med Entomol. 1993;30: 922–927. doi:10.1093/jmedent/30.5.922

37. Soni M, Khan SA, Bhattacharjee CK, Dutta P. Experimental study of dengue virus infection in Aedes aegypti and Aedes albopictus: A comparative analysis on susceptibility, virus transmission and reproductive success. J Invertebr Pathol. 2020;175: 107445. doi:10.1016/j.jip.2020.107445

38. Vazeille M, Mousson L, Rakatoarivony I, Villeret R, Rodhain F, Duchemin JB, et al. Population genetic structure and competence as a vector for dengue type 2 virus of *Aedes aegypti* and *Aedes albopictus* from Madagascar. Am J Trop Med Hyg. 2001;65: 491–497. doi:10.4269/ajtmh.2001.65.491

39. Buckner EA, Alto BW, Lounibos LP. Vertical transmission of Key West dengue-1 virus by *Aedes aegypti* and *Aedes albopictus* (Diptera: Culicidae) mosquitoes from Florida. J Med Entomol. 2013;50: 1291–1297. doi:10.1603/me13047

40. Sasaki T, Higa Y, Bertuso AG, Isawa H, Takasaki T, Minakawa N, et al. Susceptibility of indigenous and transplanted mosquito spp. to dengue virus in Japan. Jpn J Infect Dis. 2015;68: 425–427. doi:10.7883/yoken.JJID.2014.511

41. Souza-Neto JA, Powell JR, Bonizzoni M. Aedes aegypti vector competence studies: A review. Infect Genet Evol. 2019;67: 191–209. doi:10.1016/j.meegid.2018.11.009

42. Kek R, Hapuarachchi HC, Chung C-Y, Humaidi MB, Razak MABA, Chiang S, et al.

Feeding host range of *Aedes albopictus* (Diptera: Culicidae) demonstrates its opportunistic host-seeking behavior in rural Singapore. J Med Entomol. 2014;51: 880–884. doi:10.1603/me13213

43. Gimnig JE, Kolczak MS, Hightower AW, Vulule JM, Schoute E, Kamau L, et al. Effect of permethrin-treated bed nets on the spatial distribution of malaria vectors in western Kenya. Am J Trop Med Hyg. 2003;68: 115–120.

44. Reiskind MH, Lounibos LP. Spatial and temporal patterns of abundance of *Aedes aegypti* L. (*Stegomyia aegypti*) and *Aedes albopictus* (Skuse) [*Stegomyia albopictus* (Skuse)] in southern Florida. Med Vet Entomol. 2013;27: 421–429. doi:10.1111/mve.12000

45. Mogi M, Miyagi I, Abadi K, Syafruddin null. Inter- and intraspecific variation in resistance to desiccation by adult *Aedes (Stegomyia)* spp. (Diptera: Culicidae) from Indonesia. J Med Entomol. 1996;33: 53–57. doi:10.1093/jmedent/33.1.53

46. Juliano SA, O'Meara GF, Morrill JR, Cutwa MM. Desiccation and thermal tolerance of eggs and the coexistence of competing mosquitoes. Oecologia. 2002;130: 458–469. doi:10.1007/s004420100811

47. Turell MJ, Beaman JR, Tammariello RF. Susceptibility of selected strains of *Aedes aegypti* and *Aedes albopictus* (Diptera: Culicidae) to chikungunya virus. J Med Entomol. 1992;29: 49–53. doi:10.1093/jmedent/29.1.49

48. Vega-Rúa A, Zouache K, Girod R, Failloux A-B, Lourenço-de-Oliveira R. High level of vector competence of *Aedes aegypti* and *Aedes albopictus* from ten American countries as a crucial factor in the spread of Chikungunya virus. J Virol. 2014;88: 6294–6306. doi:10.1128/JVI.00370-14

49. Quan TM, Phuong HT, Vy NHT, Thanh NTL, Lien NTN, Hong TTK, et al. Evidence of previous but not current transmission of chikungunya virus in southern and central Vietnam: Results from a systematic review and a seroprevalence study in four locations. PLoS Negl Trop Dis. 2018;12: e0006246. doi:10.1371/journal.pntd.0006246

50. Nguyen-Tien T, Lundkvist Å, Lindahl J. Urban transmission of mosquito-borne flaviviruses – a review of the risk for humans in Vietnam. Infect Ecol Epi. 2019;9: 1660129. doi:10.1080/20008686.2019.1660129
