# Supplementary material for: A comparative study of dengue virus vectors in major parks and adjacent residential areas in Ho Chi Minh City, Vietnam
Source: PLoS Negl Trop Dis. 2022 Jan 12;16(1):e0010119. doi: 10.1371/journal.pntd.0010119 (PMC8789112; doi:10.1371/journal.pntd.0010119)
Supplement: Attachment — Submitted filename: PNTD-D-21-01057-patchara.docx [file pntd.0010119.s006.docx]

**PNTD-D-21-01057**

A comparative study of dengue virus vectors in major parks and adjacent residential areas in Ho Chi Minh City, Vietnam

**Abstract**

Good summary

**Intro**

Line 100: “Ae. albopictus” and A. albopicuts are asynthchronized. Please correct on the whole document (in result and discussion part).

**Method:**

*Study site:*

- Please add the vector control (both larva and adult) situation in the study sites and what actions are currently treat in the area including the resistant report (if available).
- More describe about the geographical area, people demographic and occupation that present the potential of human-vector contact.
- Good systemic design for the protocol of larva collection but the too complicate to understand overall.

*Line 150:*

- what method did you use for adult collection? Please define clearly. Do you use the swipe method for 5 mins each point? Do you have any citation by this? Where location focus to collect adults? Rewrite this section.
- Please describe how many total sampling numbers and sites per park (228) (from total 6 parks).
- IT would be better if the map of collection sites in the supplementary data; residence area and parks (with adjacent areas) are added that will image the distribution of collection.

**Results**

- Good represent of the correlation results in Figure 1 among multi couple factors (A—F).
- The results could image and understand the correlation among factors and mosquito abundance properly.
- It would be nice indicator to show percent infection rate (or the previous study) in the obtaining mosquitoes and subsequently more impact to point the risk of transmission area.
- Line 288: what is the rationale of the sampling period related to the mosquito abundance? And how to related to the transmission risk unless done by the human landing rate?

**Discussion**

- Generally, many researches showed the non-correlation of vector abundance and transmission risks. How do you explain by this issue?
- Line 337-340: “The movements of people and viruses would be less within the residential areas compared with the parks, unless large commercial activities are present; and on the whole viruses would be transmitted more efficiently and effectively in the urban parks than the residential areas.” It there any evidence to show by this sentence? This discussion part is not related to the study. Otherwise, cut this section.
- Please more discussion for *Ae. agypti* that highly contact with human and what should be more focused in term of factor correlate-transmission risk.

**Implication**

- Please highlight: what is the outstanding knowledge that obtained from this study and imply for vector control program in the area; focus location point of vector control priority, distance of insecticide spray, how to reduce the human-vector contact risk? And how to treat in the vegetable are/parks related to albopictus habitat?
- How the current larvicidal control operate in the area? That should be adjust to match with this study outcome?
