# Supplementary material for: A comparative study of dengue virus vectors in major parks and adjacent residential areas in Ho Chi Minh City, Vietnam
Source: PLoS Negl Trop Dis. 2022 Jan 12;16(1):e0010119. doi: 10.1371/journal.pntd.0010119 (PMC8789112; doi:10.1371/journal.pntd.0010119)
Supplement: Attachment — Submitted filename: Rresponses to reviewers.docx [file pntd.0010119.s008.docx]

Dear reviewers

We appreciate the time and effort that you have dedicated to providing valuable feedback on our manuscript. Your comments and suggestions are very helpful, and we agree with them. We believe that the manuscript has been much improved. We have incorporated changes to reflect your suggestions. The changes are indicated in the 'track changes' version. Our point-by-point responses to the reviewers’ comments are outlined in the attached file. Each response is in blue immediately below each reviewer’s comment (in black).

Overall, we limited our discussion about infection risk, but rather we focus on vectors. Accordingly, we deleted two paragraphs in the discussion section.

While editing the manuscript, we found some errors, and made additional corrections that were not suggested. We regret this. However, these do not affect the results and conclusion.

**Responses to reviewer 1.**

**Intro**

- Line 100: “Ae. albopictus” and A. albopicuts are asynthchronized. Please correct on the whole document (in result and discussion part).

We corrected this throughout the document. We adapted “*A. albopictus*”.

**Method:**

*Study site:*

- Please add the vector control (both larva and adult) situation in the study sites and what actions are currently treat in the area including the resistant report (if available).

We added the information with a reference: Line 136 ~

“The local dengue control programs mainly employ reactive insecticide spraying around houses of reported cases. However, this was not the case in the study area before and during the present study in the 2014 season (Fig 1). Larviciding is not a main control measure for the control programs, and it was not employed in the study area during the season.”

- More describe about the geographical area, people demographic and occupation that present the potential of human-vector contact.

We added more information and a map (Fig 1); however, we could not obtain the information on occupation. Line 113 ~

“The population in 2019 was nearly 9 million (over 8% of the total population of the country) within 2,061 km^2^, and nearly 7 million in the 19 inner districts (urban area: 283 km^2^) where we conducted the present study (Fig 1) [31].”.

- Good systemic design for the protocol of larva collection but the too complicate to understand overall.

We added supplemental figures, S1 ~ S3, to explain the sampling designs for larvae and adults.

*Line 150:*

- what method did you use for adult collection? Please define clearly. Do you use the swipe method for 5 mins each point? Do you have any citation by this? Where location focus to collect adults? Rewrite this section.

We used a sweep net for adult collection. We rewrote this section. We could not find an appropriate reference, but added supplemental figures (S 2) and a photo (Fig 2D) to visualize the sampling designs.) Line 184~ ; we did not add the paragraph here, because it is long.

- Please describe how many total sampling numbers and sites per park (228) (from total 6 parks).

We added the total number of sampling sites, 1,728 sites. Line 207

- IT would be better if the map of collection sites in the supplementary data; residence area and parks (with adjacent areas) are added that will image the distribution of collection.

We added a map (Fig 1) and supplemental figures for the sampling designs.

**Results**

- Good represent of the correlation results in Figure 1 among multi couple factors (A—F).
- The results could image and understand the correlation among factors and mosquito abundance properly.

Suggested by another reviewer, we changed the colors (red and blue) to make them clear. Fig 3

- It would be nice indicator to show percent infection rate (or the previous study) in the obtaining mosquitoes and subsequently more impact to point the risk of transmission area.

Little information was available on infection rate of mosquitoes in the city, but we added one sentenced with a reference. Line 139~

“A study conducted between 2010 and 2013 reported that the prevalence of DENV infection in *A. aegypti* was 1% in the residential area in the central part of the city [18].”

- Line 288: what is the rationale of the sampling period related to the mosquito abundance? And how to related to the transmission risk unless done by the human landing rate?

“Since *A. albopictus* and *A. aegypti* often display a bimodal peak activity [14–16],we sampled adults during the morning hours and the afternoon hours.“ Line 204..

We collected only mosquitoes that approached a collector with a sweep net, and the collector was standing at one place, and did not walk around to collect them. We think that this approach is comparable to the human landing collection method. We added this statement in the manuscript. We did not used the human landing catch method to minimize the risk of infection. Line 194 ~.

**Discussion**

- Generally, many researches showed the non-correlation of vector abundance and transmission risks. How do you explain by this issue?

We think that it is not easy to reveal the relationships between vector abundance and transmission risk. Most studies have sample size and design issues. A 50% reduction of mosquitoes might not affect human infection. In the case of malaria, a 90% of reduction might not reduce human infection, because of the differences in biology (such as lifespan) between human and mosquitoes. Yet, it is general consensus that prevalence of infection in human population increases during the rainy season when vectors become abundant. However, this is not a main scope of our study, and we limit the discussion about human infection risk and vector abundance; we deleted two paragraphs and a few sentences in the discussion related to infection risk and vector abundance. Suggested by the other reviewers. we focus on vectors, and less on the relationships between mosquito abundance and human infection. The modifications do not affect the findings and conclusion.

- Line 337-340: “The movements of people and viruses would be less within the residential areas compared with the parks, unless large commercial activities are present; and on the whole viruses would be transmitted more efficiently and effectively in the urban parks than the residential areas.” It there any evidence to show by this sentence? This discussion part is not related to the study. Otherwise, cut this section.

We deleted this paragraph.

- Please more discussion for *Ae. agypti* that highly contact with human and what should be more focused in term of factor correlate-transmission risk.

We added a few more sentences for *Ae. aegypti.* For instance, Line 426 ~.

“Although the overall density of *A. aegypti* was lower than that of *A. albopictus* in the present study, the contact between human and this mosquito species should be greater in houses, and DENV may be efficiently transmitted. “

**Implication**

- Please highlight: what is the outstanding knowledge that obtained from this study and imply for vector control program in the area; focus location point of vector control priority, distance of insecticide spray, how to reduce the human-vector contact risk? And how to treat in the vegetable are/parks related to albopictus habitat?
- How the current larvicidal control operate in the area? That should be adjust to match with this study outcome?

Responding the two comments above: We think that periodic removal of discarded containers is still the best control measure for the parks. We hope that the local dengue control programs or government will include this as a part of the park management. We also suggested reducing understory vegetation for controlling both larvae and adults. Line 470 ~

“The results suggest that periodic removal of discarded containers in the parks is important for vector control. Managing over-grown understory vegetation not only reduces the quality of larval habitats [44], but also adult mosquito resting sites [66]. The study results regarding the distances from the parks suggest that the importance of the peripheral area for vector control; however, further studies are needed to confirm this notion.”

As suggested by another reviewer, we discussed the value of park level study. Line 479 ~

“Although the comparison of vector populations among the parks was not an aim of the present study, the information should be useful for the local vector control programs. The results from the present study imply that the difference in vegetation cover may reflect on the difference in distribution and abundance of vectors among the parks. Identifying the factors related to the distribution of breeding habitats in the parks will also become valuable information for vector control.”

**Responses to Reviewer #2**

S**tudy site**

The methods was OK but need to be more clearly explained to assure the reader got the idea. Check the comment and question in the Methods section. The sample size, although not been large, but i think enough to draw the conclusion, since the results has clear pattern (at least for the overall results of the 6 parks)

We added more information in the section, and a map (Fig 1). Line 113~. Responses to the comments in the PDF file are below.

**Results**

The results, mostly, are as expected that A. albopictus was more vegetation dependent (park, outdoor) and A. aegypti was human-associated dependent (indoor, artificial containers).  The results presented were based on the total results from all the six parks analyzed in the city, however it will be more clear and more interesting if the authors can show the one or two most important results of each park (not only the overall results after mixing the data from 6 parks) to see whether the pattern discussed are more or less in all the parks. It will be interesting to see how the pattern of each park on the preference of park vs residential area, or indoor vs outdoor for both A. aegypti and Ae. albopictus.

We agree an individual park level study will be worthwhile and should provide more valuable information. We added a comment in the implication section.

“Although the comparison of vector populations among the parks was not an aim of the present study, the information should be useful for the local vector control programs. The results from the present study imply that the difference in vegetation cover may reflect on the difference in distribution and abundance of vectors among the parks. Identifying the factors related to the distribution of breeding habitats in the parks will also become valuable information for vector control.”)

The boxplot graph in figure 2 show very few information, mostly similar between the species comparison. Even, can draw contradictory conclusion between the figure and the tables. If all the information covered by the tables presented, then better to remove figure 2.

We deleted the box plot. Instead, we inserted averages, medians and ranged in the Table7 and 8.

In tables presented there were writing inconsistency for the value of references, some were '1' and the others were '1 ref' or '1 (ref)'.
Please also respond to the comments in the Results section.

We replaced “1 “ and “1 ref” with “1 (ref)”.

**Discussion**
The conclusion about the preference of sites and habitats are OK. However, the dengue transmission risk should not only considered by the abundance of vector species and its site habitat preferences, but also the abundance of host that they may encounter (host population in residential area are clearly higher than in park since the site is their home, in the morning peoples are still in their houses before work, while afternoon most people are comeback to their houses. Although some may visit parks, but it may only less than 30% of population. It is good if authors can show data about density of host in parks vs residential area, so the conclusion will have bases. Also, the natural presence of endo symbiont such as Wolbachia spp. in A. albopictus but not in A. aegypti, that may affect the capacity vectorial of A. aegypti several folds higher than A. albopictus. 

We limited our argument on transmission risk based on the abundance of A. albopictus. This was suggested by the other reviewer. We deleted two paragraphs in the discussion related to this.

Earlier studies did not detect *Wolbachia* spp in A. aegypti; however, there are some recent reports natural *A. aegypti* populations infected with the bacteria. Therefore, we would like to avoid a discussion related to the effects of Wolbachia.

1: Kulkarni A, Yu W, Jiang J, Sanchez C, Karna AK, Martinez KJL, Hanley KA,

Buenemann M, Hansen IA, Xue RD, Ettestad P, Melman S, Duguma D, Debboun M, Xu J.cWolbachia pipientis occurs in Aedes aegypti populations in New

Mexico and Florida, USA. Ecol Evol. 2019 Apr 26;9(10):6148-6156. doi:

10.1002/ece3.5198. PMID: 31161026; PMCID: PMC6540660.

2: Carvajal TM, Hashimoto K, Harnandika RK, Amalin DM, Watanabe K. Detection of

Wolbachia in field-collected Aedes aegypti mosquitoes in metropolitan Manila,

Philippines. Parasit Vectors. 2019 Jul 24;12(1):361. doi:

10.1186/s13071-019-3629-y. PMID: 31340862; PMCID: PMC6657204.

Overall conclusion was OK, but need to add some discussion on the mentioned aspects.

The Methods section should be write more clearly to not confuse readers whether one methods used for both park or residential, or only true for either park or residential area. The authors please check the comments in the pdf manuscript in method section and clarify according to the question.

We rewrote the method section and added some figures (Figure 1 and 2, S1, S2 and S3) to explain the details of sampling methods. Also see responses to the comments in the PDF.

This study is interesting, and give more insight to common readers, as well as officers who performed the vector control programs, emphasize of common knowledges about preference site and habitats of A. aegypti and A. albopictus with clear data. It is worth to published with a minor revision in method section (to be clearer) and additional discussion on the risk of viral transmission, especially dengue.

We added supplemental figures to explain the sampling designs better. We also added more implications for the vector control.

**Responses to the comments on the PDF file**

Line 131: Were this categories true for park or residential area? or both?

We categorized habitats in both parks and residential areas. We added a sentence: Line 177.

We searched for larvae not only outdoor, but also inside of buildings in the parks. Line 153.

Line 138: This part talking about structured sampling in residential area. It is not clear whether the categories in previous paragraph were also true for this residential area, since it was mention the section of larval sampling in parks.

We added a few sentences to describe the details of the sample method in the residential areas, including a sentence for the habitat category Line 176.

Line 148: I think the larval sampling methods need to explain clearer details for park and residential area in separate paragraphs and not to mix explanation for both, so reader can understand how the sampling methods were performed in parks and in residential area

We have separate paragraphs for the parks (Line 148~) and the residential areas (Line 165~)

Line 153: Is this mean each park have 8 sampling sites for adults? or it means there are 48x 6 sampling points for all 6 parks?

Each park has 288 sampling points = 48 sites x 6 sampling points. We indicated it in the sentence (Line 190), and provided a supplemental figure (S2) for the sampling design.

Line 154: How many days adults samplings were performed?

2 days: 2 sampling periods (morning and afternoon for each day. We added a sentence to describe this. Line 204

Line 155: It is means there were actually 6x6 sampling points for total?

12 sites x 6 sampling points = 72 sampling points for each sampling period. Line 191.

Line 158: So the total time for cycles in one sampling points was 10 minutes include sampling and pause period?

Yes. We added the information in Line 198.

Line 164: How many collectors actually performed the sampling for 288 points within 2 days?

Twelve field workers were involved in the survey for each park (8 collectors for each residential area). We indicated the number of collectors in Line 199 for sampling adults at the park and line 219 for the residential areas. We also indicated the number of field workers involved in the larval survey and in Line 162 for the parks and 181 for the residential area.

Line 166: What is the reason why for adults sampling only 16 transect line used, instead of 20 as in larval sampling? why did not adjust to match larval and adults sampling points?

The adult sampling required more time for each house than the larval sampling, and we wanted to reduce the traveling time between the transect line or house. We inserted the reason in the text. Line 211

Table 1: Is the proportion in percentage?

Yes. We added (%) to the table title.

Table 1: Are all of 1988 habitats filled with water at the time of the survey/observation? or all habitats were counted regardless the presence or water?

Yes, all of 1988 habitats were filled with water. We modified the table title.

Table 2: please also make clear is this 'proportion' means 'percentage'?

We added “%” to the table title.

Table 2: Could you explain how can be any indoor habitats in a park?

There were a few buildings, and sheds in the parks. We added the information in the text. Line 154

Table 3: Have you defined what is geographical area in the method section. It is suddenly appear in results section with no introduction what it means by your operational definition?

“Geographical area” is the area including both each park and corresponding adjacent residential area. We added a sentence to provide the information in the method section. Line 234.

Line 223: Is the potential habitat means habitat (any types of water containers) filled with water or include habitats without water inside?

“ the potential habitat means “filled with water”. We indicated “potential water holding habitats…”, Line 150

Table 4: What do the '1 ref' mean? why in other cells showing the PR value of PR only notated '1'? Could you give some short description on how you calculate the PR? and how people interpret the adjusted and unadjusted values?

“1” and “1 ref” are the baseline reference. They are same, but we mixed “1” “1 ref” and 1 (ref)” among the tables. We use the same terminology “1 (ref)” throughout the manuscript. PR means a ratio between the prevalence of a target habitat and that of the other habitats. A prevalence is a proportion of positive sites or sites mosquito larvae were present. PR is estimated based on the prevalence of the other habitats considering as the baseline (1 (ref)). A PR in the tables might be slightly different from the observed prevalence ratio, because the PR is estimated from a regression model. An unadjusted PR is estimated from a single logistic regression model, and an adjusted PR is estimated from a multiple regression model with confounding variables. If a PR is 0.55, this may indicate that the occurrence of larvae in the target habitat would be 45% less compared to the other habitats. The terminology has been mainly used in epidemiology; however, the use is getting popular in medical entomology.

**Responses to reviewer 3**

probably should define “urban parks” very early in the manuscript.  This could be very different from playground style with groomed landscape to simply native plants in an otherwise Metropolitan/concrete focus.    Are there large trees or mostly flowers?  Is it basketball courts or open grassy areas?  Providing some “visual” imagery of what type of urban park you are referring to would assist the readers in understanding how this might look different from a residential area (sub-urban presumably).

We added photos (Fig 2A for park and 2B for residential area) and more information for the parks. Line 113 ~ 123.

An additional general comment is the difficulty in reading the text as so many comparisons were made but it wasn’t always clear which items were being compared, presented, or discussed.  Please consider reviewing the manuscript (throughout) to make certain each statement such as “urban area has more prevalence” is clear regarding what is “more” in the comparison.

We tried to make them clear throughout the manuscript.

Specific:
Line 52:  aren’t albos dusk/dawn biters over general day biting?  Please provide references for each species as a daytime biter.

We added more information with references for this statement in the introduction. Line 80 ~81).

“Although they often display a bimodal peak activity during the morning and late afternoon [14–16], the peak hours may vary depending on environmental conditions [17].”

Line 60:  is there currently no vector control in parks?  If some, what type are you referring to here?  (ie. truck based spraying or trash removal?, etc.)

There was not a systematic vector control activity in the parks, and the current control programs still do not consider parks.

Line 77:  not clear how transitions toward urban areas results in more seropositivity in rural areas.  Might consider revising this sentence for clarity.

We added “infected with DENV..” to the sentence for clarity; “the high seropositive rate in rural areas is likely due to an increase of mobile populations infected with DEMV from urban areas, ….” Line 67 ~ 69

Line 109:  are more natural egg-laying sites important when you’ve stated that these mosquitoes readily lay eggs in discarded trash?   Also, when referring to “breeding sites”, do you really mean “egg-laying sites” (which is really the correct term even though breeding site is colloquially used).   Might consider using the proper term.

In the first appearance of “breeding site”, we change the phrase to “larval breeding site” to be more specific (line 105). Likely these sites are egg-laying sites. Since we did not observe oviposition, but larvae, we prefer “larval breeding site”. We checked several publications, and we confirmed “breeding site” is commonly used.

Lines 121-124:  might consider describing the ecology of the parks under study.
We added more information of the parks in the study site section. Line 116 ~123. We also added a photo (Fig. 2A).

Lines 180-181:  if a site had a single target mosquito on a single collecting trip, would this site have been considered positive?   Another site could have 100 target mosquitoes on a single trip and yet would be considered “positive” as the first site?  Seems the plus / minus approach might not indicate significant differences among the sites, correct?   How does the study design account for numeric differences?

When a target mosquito species was present at a habitat, the site was considered as positive regardless the number of larvae. Even though 100 mosquito individuals were present at the site, or 1 individual was present, the site was considered as positive. It is not easy to quantify the number of larvae at each site because of diverse habitat types. There is not a standard sampling method for all habitat types. We added one reference. The study challenged this issue, but the method still does not apply to various habitat types [36].

To analyze the qualitative data (0 or 1), we used corresponding analysis and logistic regression model for presence and absent data. These are adequate statistical methods to deal with qualitative data with binomial distribution.

To make the argument clear, we rewrote the statistical analysis section.

Figure 1 – the light coloring of the text in each graph makes them difficult to read.

We corrected this problem adapting red and blue colors.

Lines 228 and near – please use caution when describing something as having a higher or lower presence as sometimes, it is not clear if you mean higher in that container versus other container types or higher in that container type for one species versus the other species.  Please make sure each comparison is clear.

We corrected this issue throughout the manuscript.

Table 4 – text too small to read

We made it bigger using landscape format. .

Line 303-304- please provide a reference for this statement

We added a sentence and a reference (line 397~399 and [43]

“The study in São Paulo also found a positive association of *A. albopictus* with vegetation in a much larger urban park which includes a remaining natural forest [43].”

Line 330-331 --  this line is a bit misleading as it doesn’t truly assess the time this species bites the most.  It just compares one specific morning time versus one specific afternoon time.  Since other times were not considered (ie – dusk), the sentence should be clarified.

We deleted this paragraph, because we did not collect mosquitoes throughout the day. We also limited the discussion about infection risk.

Line 343 – this is a bit confusing as in the results, it was noted that both species were preferentially found in the outdoor sites.   Please clarify.

In the result section, we mentioned “the density of *A. aegypti* in the residential areas was significantly less in the outdoor habitats compared with the indoor habitats” Line 364~365). However, the difference in density between the habitats was not statistically significant for *A. albopictus* (Table 8). When the two species were combined, the density was significantly lower in the outdoor habitats in the residential areas.”

Line 345-346 – this sentence seems out of place in this paragraph.

We deleted this sentence.

Line 372 – this statement is only partially accurate.  Albopictus is only more susceptible to ECSA strains of CHIKV with a specific mutation at position 226.  Other strains of CHIKV are typically better vectored by aegypti.  Please correct this sentence.

We removed the sentence and added more information of the E1-226V strain. Line 451 ~ 456

Lines 373-375 – this sentence seems unnecessary here.  Suggest deleting it
We rewrote the entire paragraph to suggest potential transmission risk of other viral diseases by *A. albopictus*. Line 448 ~456

Line 389-90. – this sentence, while likely a good idea, is not based on anything presented in this paper.  Suggest deleting it.

We deleted the sentence.
